# Protopanaxadiol manipulates gut microbiota to promote bone marrow hematopoiesis and enhance immunity in cyclophosphamide‐induced immunosuppression mice

**DOI:** 10.1002/mco2.222

**Published:** 2023-02-23

**Authors:** Yuru Cao, Ben Liu, Wenzhen Li, Feng Geng, Xue Gao, Lijun Yue, Huiping Liu, Congying Liu, Zhenguo Su, Junhong Lü, Xiaohong Pan

**Affiliations:** ^1^ School of Pharmacy Binzhou Medical University Yantai China; ^2^ Yantai Affiliated Hospital of Binzhou Medical University Yantai China; ^3^ Shanghai Advanced Research Institute Chinese Academy of Sciences Shanghai China; ^4^ Jinan Microecological Biomedicine Shandong Laboratory Jinan China

**Keywords:** chemotherapy, gut microbiota, immunity, immunomodulator, immunosuppression, protopanaxadiol

## Abstract

Protopanaxadiol (PPD) has potential immunomodulatory effects, but the underlying mechanism remains unclear. Here, we explored the potential roles of gut microbiota in the immunity regulation mechanisms of PPD using a cyclophosphamide (CTX)‐induced immunosuppression mouse model. Our results showed that a medium dose of PPD (PPD‐M, 50 mg/kg) effectively ameliorated the immunosuppression induced by CTX treatment by promoting bone marrow hematopoiesis, increasing the number of splenic T lymphocytes and regulating the secretion of serum immunoglobulins and cytokines. Meanwhile, PPD‐M protected against CTX‐induced gut microbiota dysbiosis by increasing the relative abundance of *Lactobacillus*, *Oscillospirales*, *Turicibacter*, *Coldextribacter*, *Lachnospiraceae*, *Dubosiella*, and *Alloprevotella* and reducing the relative abundance of *Escherichia‐Shigella*. Importantly, PPD‐M lost the ability to promote bone marrow hematopoiesis and enhance immunity when the gut microbiota was depleted by broad‐spectrum antibiotics. Moreover, PPD‐M promoted the production of microbiota‐derived immune‐enhancing metabolites including cucurbitacin C, l‐gulonolactone, ceramide, DG, prostaglandin E2 ethanolamide, palmitoyl glucuronide, 9R,10S‐epoxy‐stearic acid, and 9′‐carboxy‐gamma‐chromanol. KEGG topology analysis showed that the PPD‐M treatment significantly enriched the sphingolipid metabolic pathway with ceramide as a main metabolite. Our findings reveal that PPD enhances immunity by manipulating gut microbiota and has the potential to be used as an immunomodulator in cancer chemotherapy.

## Graphical abstract

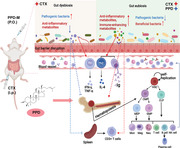

## 1 INTRODUCTION

Chemotherapy is the main method for the treatment of cancers, especially for patients with moderate and advanced metastatic tumors. However, chemotherapy agents, such as cyclophosphamide (CTX), tend to cause significant immunosuppression, including myelosuppression, leukopenia, anemia, and gastrointestinal mucosal barrier damage.^1^, ^2^, ^3^ The emergence of immunosuppression will lead to a relative reduction in the use of chemotherapy drugs, reduce the therapeutic effect, and result in infections and mortality in cancer patients. Therefore, discovering an effective immunomodulator to alleviate immunosuppression is of great significance to reduce the side effects and enhance the efficacy of chemotherapy drugs.

Ginsenosides, the major active ingredients found in ginseng and other plants of the genus Panax, are very popular for their antioxidant, strengthening the immune system and many other activities.^4^, ^5^ Protopanaxadiol (PPD) is a major active metabolite from PPD‐type ginsenosides and possesses pleiotropic anticancer activities in many cancers.^6^, ^7^, ^8^ Enhancing immunity is one of the main functions of ginsenosides. Whether PPD also regulates immunity has rarely been studied. Only one article reported that PPD significantly enhances the antilung cancer effect of CTX. In this process, PPD increases the spleen index, peripheral white blood cell (WBC) count, natural killer cell activity, and the levels of interleukin‑2 (IL‑2) and interferon‑γ (IFN‑γ) in CTX‑treated tumor‑bearing mice,^9^ suggesting that PPD may ameliorate the immunosuppression induced by CTX. However, the mechanism by which PPD enhances immunity remains unclear.

The gut microbiota plays an important role in maintaining immune homeostasis.^10^, ^11^ Under normal physiological conditions, the gut microbiota is in a dynamic balance with the immune system, which plays a critical role in maintaining human health.^12^ Disruption of gut microbiota will upset this balance, and the immune system will be compromised, leading to inflammation and related diseases.^10^, ^13^ Chemotherapeutic agents often disrupt the ecological balance of gut microbiota, lead to the overexpression of certain pathogenic bacteria, and promote the occurrence of chronic inflammation and immunosuppression.^14^, ^15^ Therefore, the concurrent use of gut microbiota modulators in cancer chemotherapy is considered to be a promising strategy. Many active ingredients in plants have been found to regulate gut microbiota and improve immunity.^16^, ^17^, ^18^, ^19^, ^20^ Whether PPD can regulate gut microbiota has not been reported.

Here, we investigated the protective effects of PPD on the immune system and gut microbiota in CTX‐induced immunosuppressed mice. 16S‐rRNA sequencing and untargeted metabolomic analysis were employed to reveal the mechanism by which PPD enhances immunity. It was confirmed that PPD promoted bone marrow hematopoiesis and enhanced immunity by regulating gut microbiota to ameliorate the immunosuppression induced by CTX.

## 2 RESULTS

### 2.1 PPD improves immune function in immunosuppressed mice

**FIGURE 1:**
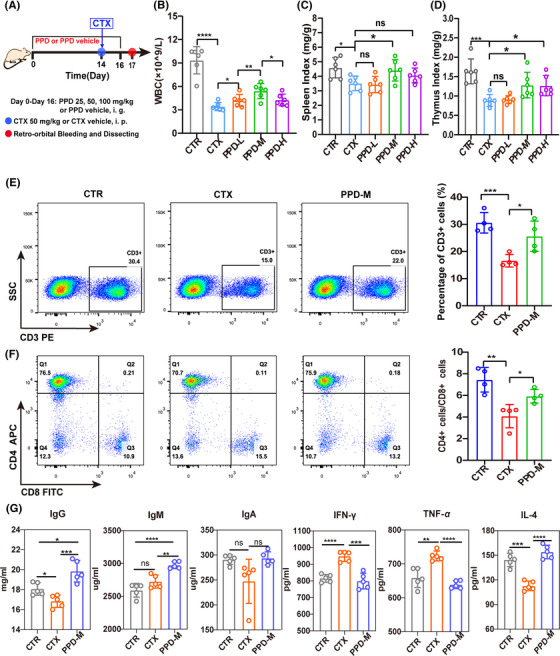
Effects of PPD on immunity in immunosuppressed mice. (A) Mice were intragastrically treated with different concentrations of PPD (PPD‐L: 25 mg/kg, PPD‐M: 50 mg/kg, PPD‐H: 100 mg/kg) for 16 days, and the CTR group and CTX group mice were intragastrically administered PPD vehicle. Intraperitoneal injection of CTX (50 mg/kg) was performed on day 14 (CTR group mice with CTX vehicle). Blood was collected from the retro‐orbital venous plexus and mice were sacrificed on day 17. (B) Changes of WBCs in the peripheral blood of mice in each group. (C) Spleen index of mice in each group. (D) Thymus index of mice in each group. (E) The expression of CD3^+^ T lymphocytes in the mouse spleen. (F) The expression of CD4^+^ and CD8^+^ T lymphocytes in the mouse spleen. (G) The levels of IgG, IgM, IgA, IL‐4, IFN‐γ, and TNF‐α in serum. (*n* = 6, *p < 0.05, **p < 0.01, ***p < 0.001, “ns” means no significant difference.)

ICR mice were intraperitoneally injected with CTX to construct an immunosuppressive model (Figure S1). The effects of PPD on immune function in the immunosuppressed mice were evaluated by splenic lymphocyte subsets, inflammatory factors, immunoglobulins, spleen, and thymus. As shown in Figure 1A, mice were prophylactically treated with different concentrations of PPD for 14 days before CTX injection. On the 17th day, the blood of the fundus venous plexus, right femur, thymus, and spleen of mice was collected for the following experiments. The obtained results showed that, compared with the control (CTR) group, peripheral blood WBCs were significantly reduced in the CTX group, PPD significantly ameliorated this reduction, and the medium dose of PPD (50 mg/kg, PPD‐M) had the best effect (Figure 1B). Similarly, the spleen index and thymus index of mice in the CTX group significantly decreased, PPD significantly reversed the spleen index and thymus index depression caused by CTX, and PPD‐M had the best effect (Figures 1C and D, Figure S2). Here, PPD‐H (100 mg/kg) is less effective than PPD‐M in enhancing immunity, which may be due to certain cytotoxicity of higher concentration of PPD‐H. Our previous studies have found that PPD‐H, but not PPD‐M, has significant antitumor effects.^21^ Therefore, PPD‐M was used as an immunomodulator to treat immunosuppressed mice in the following experiments.

Flow cytometry results showed that the number of CD3^+^ T lymphocytes and the ratio of CD4^+^/CD8^+^ T lymphocytes in the spleen were significantly decreased in the CTX group, while PPD‐M treatment significantly reversed the decrease (Figures 1E and F). Serum immunoglobulin and cytokine levels were detected by ELISA. As shown in Figure 1G, the serum levels of IL‐4 and IgG were significantly decreased after CTX treatment, while PPD‐M treatment significantly increased their levels. Conversely, the contents of IFN‐γ and TNF‐α were clearly increased after CTX treatment, whereas PPD‐M reduced them to the control level. In addition, the IgM level in the PPD‐M group was significantly higher than that in the CTX group.

The HE staining analyses of the spleen and thymus of mice are shown in Figure S3. Compared with the CTR group, the splenic capsule thickness of the CTX group was uneven, the number and volume of white pulp were decreased, and the boundary between white and red pulp was unclear. However, in the PPD‐M group, these symptoms were improved, and a diffuse distribution of extramedullary hematopoietic cells was observed in the red pulp. The thymus volume of mice in the CTX group was reduced and the medulla structure was unclear. Although the structure of the thymus was also unclear in the PPD‐M group, the thymus volume was normal. The abovementioned results demonstrate that PPD can restore the impaired immune function induced by CTX in immunosuppressed mice.

### 2.2 PPD ameliorates leukopenia by promoting bone marrow hematopoiesis

Chemotherapy is known to target bone marrow cells to induce leukopenia, and its recovery may also occur through bone marrow hematopoiesis.^22^ The number of bone marrow nucleated cells can reflect the strength of bone marrow hematopoietic function. Therefore, bone marrow histopathology and flow cytometry were used to analyze the effect of PPD on bone marrow hematopoiesis in immunosuppressed mice. A flow cytometry gating strategy was used to identify hematopoietic stem and progenitor cells (HSPCs), common lymphoid progenitors (CLPs), common myeloid progenitors (CMPs), granulocyte‐macrophage progenitors (GMPs), and megakaryocyte‐erythrocyte progenitors (MEPs)^23^ (Figure S4).

**FIGURE 2:**
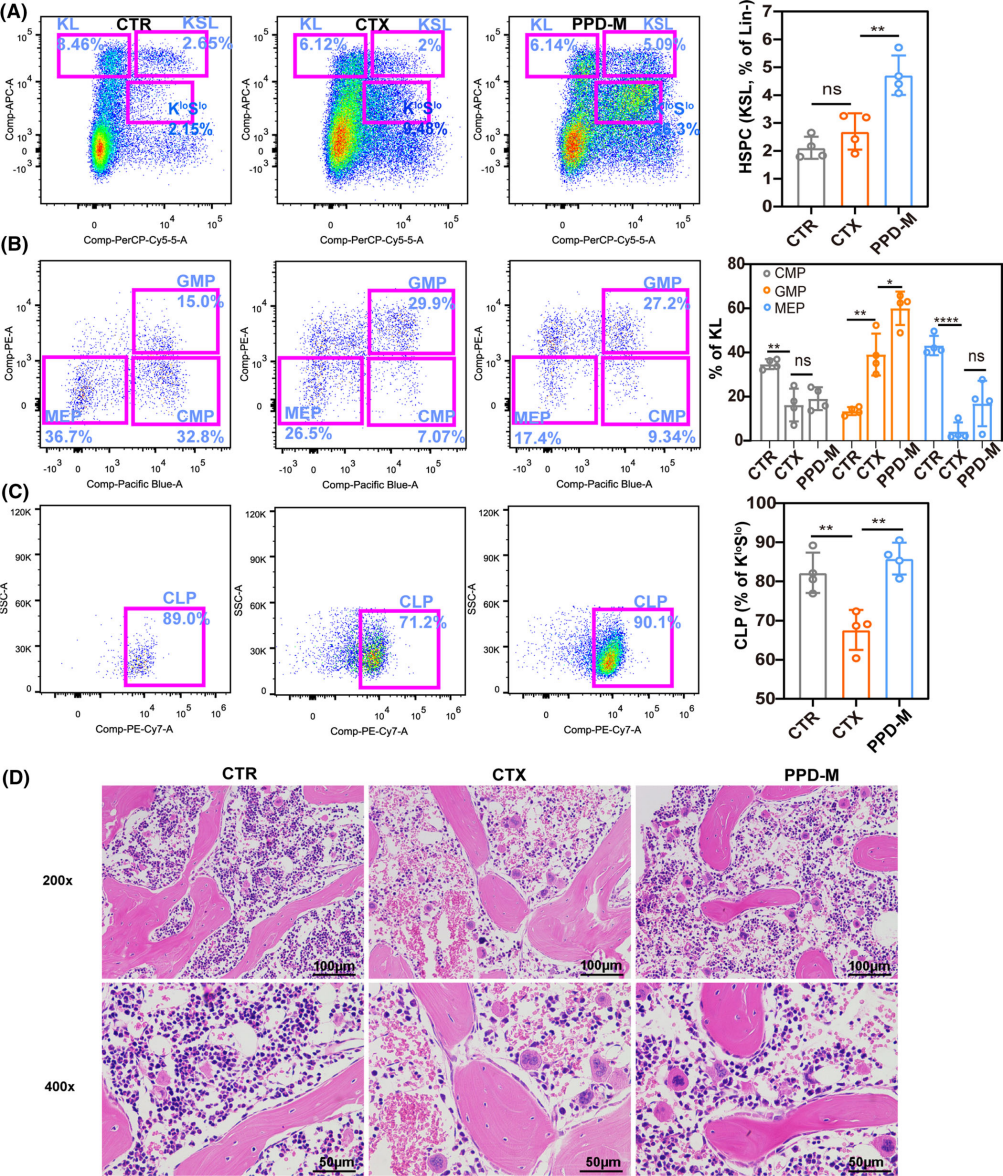
PPD improves bone marrow suppression induced by CTX in mice. (A) Changes in bone marrow hematopoietic stem and progenitor cells (HSPCs, KLS^+^) in the CTR, CTX, and PPD‐M groups. (B) and (C) Changes in bone marrow hematopoietic progenitor cells (CMPs, GMPs, MEPs, and CLPs) in the CTR group, CTX group and PPD‐M group. (D) HE staining of mouse femur tissue. (Data are expressed as the mean ± SD. *n* = 4, *p < 0.05, **p < 0.01, ****p < 0.0001, “ns” means no significant difference.)

Flow cytometry results showed that PPD‐M treatment significantly increased the number of HSPCs compared with those in the CTR and CTX groups (Figure 2A). Moreover, CTX could significantly reduce CMP, MEP, and CLP levels, among which PPD‐M clearly reversed the CLP reduction caused by CTX (Figures 2B and C). However, the GMP level in the CTX group was significantly higher than that in the CTR group, which may be a temporary increase from hematopoietic stress,^24^ and PPD‐M treatment further enhanced the level of GMPs. HE staining analysis of mouse bone marrow is shown in Figure 2D. Compared with the CTR group, nucleated cells in the bone marrow of mice in the CTX group were significantly reduced, and a large area of hemorrhage was observed, while these phenomena were improved in the PPD‐M group. These results suggest that PPD promotes HSPC self‐renewal and differentiation in immunosuppressed mice.

### 2.3 PPD improves the composition and structure of gut microbiota in immunosuppressed mice

Chemotherapy is typically associated with dysbiosis of the gut microbiota.^15^ To study whether PPD could prevent CTX‐induced gut microbiota dysbiosis, we performed high‐throughput gene sequencing of 16S rRNA from the fecal bacteria of mice. The results of the dilution curves of the Chao diversity index and Shannon diversity index indicated that most bacterial diversity and many new phylotypes could be captured (Figure S5). To determine the gut microbiota richness and diversity, the ACE, Chao, Shannon, and Simpson indices were used to evaluate the *α*‐diversity. As shown in Table 1, CTX reduced the richness and diversity of gut microbiota with lower ACE, Chao, and Shannon indices and a higher Simpson index. Of note, PPD reversed this phenomenon. These results indicate that PPD has a regulatory effect on the gut microbiota and increases the richness and diversity of intestinal bacteria.

**TABLE 1:** α‐Diversity indices of gut microbiota in each group

Groups	ACE	Chao	Shannon	Simpson
CTR	425.89 ± 51.48	434.47 ± 59.55	3.98 ± 0.31	0.05 ± 0.02
CTX	390.95 ± 94.30	395.90 ± 102.48	3.57 ± 0.61	0.10 ± 0.08
PPD‐M	424.39 ± 69.32	430.25 ± 73.75	3.95 ± 0.20	0.05 ± 0.01

ACE (Abundance‐based Coverage Estimator) and Chao indices reflect community richness, and the larger the value, the higher the community richness. Shannon (Shannon‐Wiener index) and Simpson (proposed by *Edward Hugh Simpson*) indices represent community diversity. The higher the Shannon value, the higher the community diversity. Conversely, the lower the Simpson value, the higher the community diversity.

**FIGURE 3:**
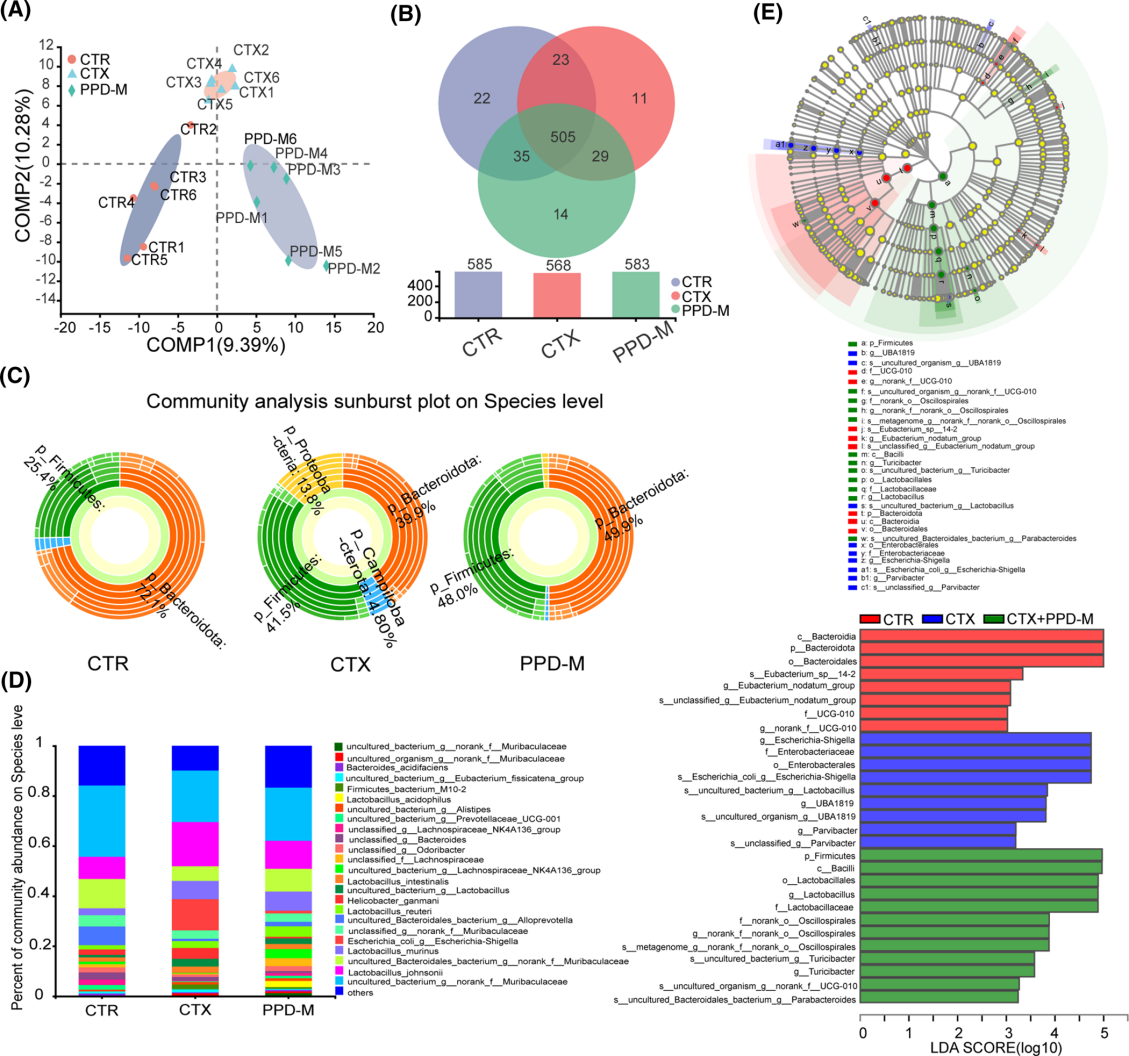
Effects of PPD on gut microbiota composition and structure in immunosuppressed mice. (A) PLS‐DA analysis (OTU level). (B) Venn diagram (similarity and difference in bacterial compositions in different experimental groups at the OTU level). (C) Community analysis sunburst plot at the species level (the species composition at the domain, kingdom, phylum, class, order, family, genus, and species levels is displayed from the inner circle to the outer circle). (D) Community bar plot analysis at the species level (the ordinate is the percentage of species in the sample, the color of the column represents different species, and the length of the column represents the size of the percentage of the species). (E) LEfSe (LDA effect size, LDA > 3) analysis of species with significant differences between CTR, CTX, and PPD‐M (the greater the LDA score is, the greater the impact of species abundance on the differential effect).

Partial least squares discriminant analysis (PLS‐DA) was used to compare the similarity between groups and showed that the mouse samples of the CTR group, CTX group and PPD‐M group could be clearly distinguished and clustered into three groups (Figure 3A), indicating that the gut microbiota composition of the three groups of mice had significant differences. The proportions for shared and unique OTUs are shown in a Venn diagram (Figure 3B). There were 505 shared OTUs among the three groups. The number of unique OTUs in the CTR, CTX, and PPD‐M groups was 22, 11, and 14, respectively. Multilevel sunburst maps at the species level showed that the dominant bacteria in the CTR group were *Firmicutes* and *Bacteroidota*. CTX treatment decreased the abundance of *Bacteroidota* and increased that of *Firmicutes*, *Proteobacteria* and *Campilobacterota*. Interestingly, PPD‐M treatment reversed the increase in *Proteobacteria* and *Campilobacterota* caused by CTX and restored the proportion of dominant bacteria to CTR group levels (Figure 3C). The community bar diagram showed that CTX and PPD had different influences on the relative abundance of microbial community composition, and the PPD group showed the advantage of having a closer evolutionary relationship with the CTR group (Figure 3D). These results indicate that PPD regulates the structure and composition of gut microbiota and reverses CTX‐induced gut microbiota dysbiosis.

To identify the key phylotypes and biomarkers of gut microbiota among the three groups, LEfSe (LDA > 3, *p* < 0.05) analysis was performed (Figure 3E). According to the analysis results, *p_Bacteroidota, c_Bacteroidia*, and *o_Bacteroidales* displayed high LDA scores, showing that they were abundant OTUs in the CTR group. In the CTX group, *g_Escherichia‐Shigella*, *f_Enterobacteriaceae*, and *o_Enterobacterales* exhibited higher scores, which indicated that they were significantly affected by CTX. In the PPD‐M group, *p_Firmicutes*, *c_Bacilli*, *o_Lactobacillales*, *f_Lactobacillaceae*, and *g_Lactobacillus* were significantly enriched, and *o_Oscillospirales*, *g_Turicibacter*, and *g_Parabacteroides* were also abundant. Thus, CTX leads to an increase in pathogenic bacteria, while PPD can enhance the abundance of beneficial bacteria, especially *Lactobacillus*. In general, these results indicate that PPD intervention ameliorates the imbalance of gut microbiota induced by CTX and promotes the proliferation of beneficial bacteria.

### 2.4 Gut microbiota depletion impairs HSPC self‐renewal and immune function recovery

**FIGURE 4:**
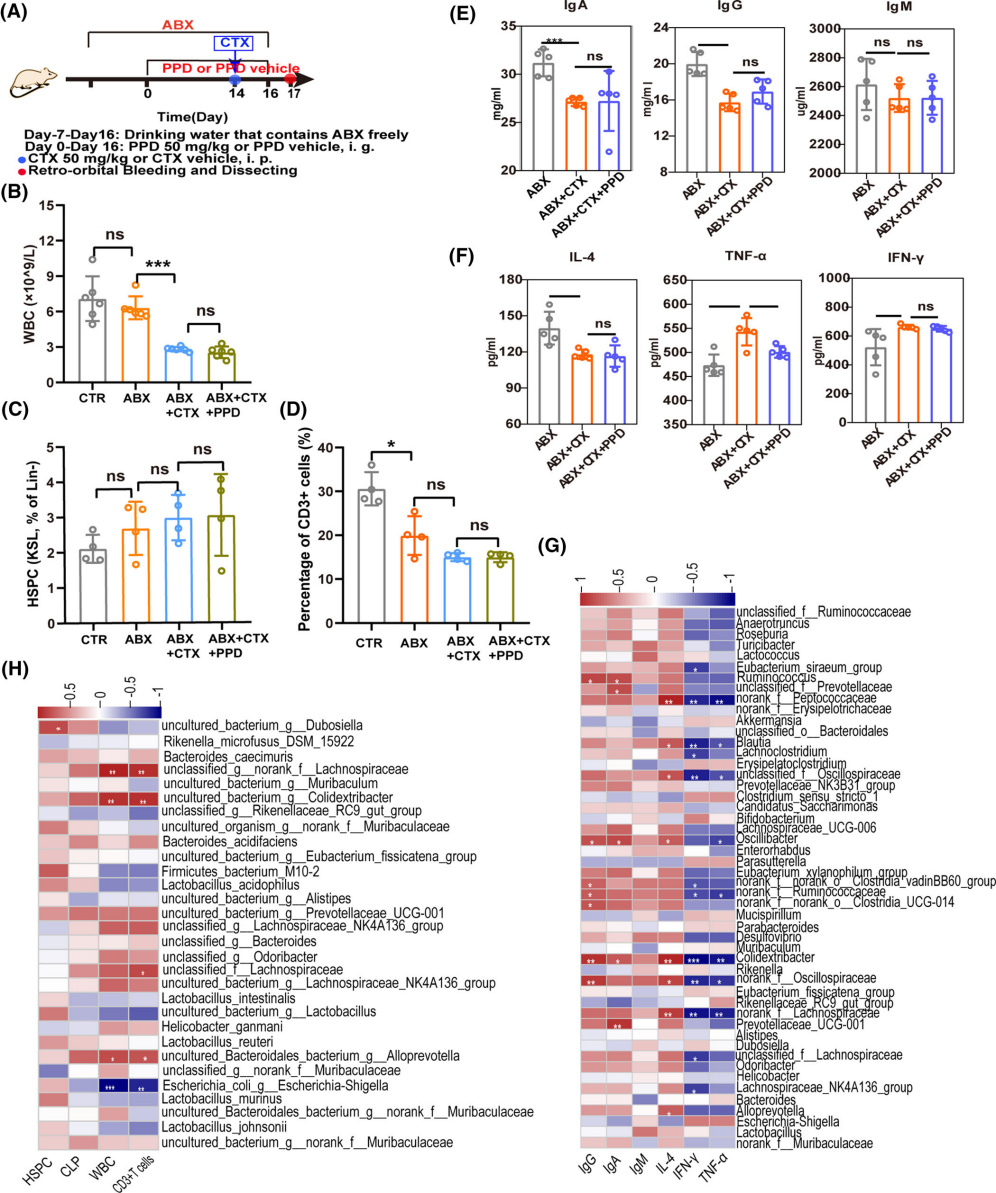
Correlation between gut microbiota and PPD enhancing immunity in immunosuppressed mice. (A) Antibiotic‐treated mouse protocol. In the first 7 days of the experiment and throughout the experiment, broad‐spectrum antibiotics were freely drunk by mice (CTR group was free to drink sterile water). The mice in the ABX+CTX+PPD group were intragastrically treated with PPD (50 mg/kg) for 16 days, and the CTR group, ABX group and ABX+CTX group mice were intragastrically administered PPD vehicle. Intraperitoneal injection of CTX was performed on day 14 (CTR group and ABX group mice with CTX vehicle). Blood was collected from the retro‐orbital venous plexus and mice were sacrificed on day 17. (B–D) Effects of PPD on WBCs (B), HSPCs (C), spleen CD3^+^ T cells (D), IgG, IgA and IgM (E), IL‐4, TNF‐α, and IFN‐γ (F) in antibiotic‐treated mice (data are expressed as the mean ± SD. *n* = 4–6, *p < 0.05, **p < 0.01, ***p < 0.001, “ns” means no significant difference). (G) Spearman correlation heatmap at the genus level. (H) Spearman correlation heatmap at the species level. Squares in red (positive correlation) or blue (negative correlation) with a white asterisk (*) indicate significant correlations with *p* < 0.05 measured by the Spearman parametric correlation test.

To determine whether the gut microbiota plays a key role in PPD promoting bone marrow hematopoiesis and restoring immune functions, broad‐spectrum antibiotics (ABX) were used to deplete the microbiota in mice (Figure 4A). In this model, the fecal bacteria of mice significantly decreased compared with the normal group (Figure S6). Interestingly, in ABX‐treated mice, PPD lost the ability to increase the number of WBCs and HSPCs (Figures 4B and C). Similarly, PPD could not reverse the decrease in CD3^+^ T cells, IgA, IgG, and IL‐4 caused by CTX (Figures 4D–F), and IFN‐γ did not return to the control level after PPD treatment (Figure 4F). These results indicate that PPD promotes bone marrow hematopoiesis and enhances immunity by regulating the gut microbiota, and the gut microbiota plays an indispensable role in the immune regulation of PPD.

Furthermore, Spearman correlation analysis was performed to evaluate the potential relationship between immune parameters and the major microbiota. As shown in Figure 4G, many bacteria were positively correlated with immunoglobulins and negatively correlated with inflammatory factors. Specifically, *Colidextribacter, f_Oscillospiraceae*, and *Oscillibacter* were significantly positively correlated with IgG, IgM, and IL‐4 and negatively correlated with IFN‐γ and TNF‐α. In addition, *f_Peptococcaceae*, *Blautia*, and *f_Lachnospiraceae* were positively related to IL‐4 and negatively correlated with IFN‐γ and TNF‐α. Moreover, *f_Lachnospiraceae, g_Colidextribacter, g_Dubosiella*, and *g_Alloprevotella* were significantly positively correlated with HSPCs, WBCs, and CD3^+^ T cells, while *g_Escherichia‐Shigella* was negatively correlated with WBCs and CD3^+^ T cells (Figure 4H). These results further confirm that the gut microbiota plays an important role in PPD regulation of immunity.

### 2.5 PPD promotes the production of immune‐related fecal metabolites

Intestinal metabolites are produced through the interaction between gut microbiota and host, which are essential for the maintenance of immune homeostasis.^25^ We hypothesized that the potential mechanisms by which PPD improves immune functions are via the secondary metabolites generated by microbial metabolism in the gut. Then, untargeted metabolomic analysis of fecal samples was performed using liquid chromatography‒mass spectrometry (LC‒MS) in the CTR, CTX, and PPD‐M groups. The PLS‐DA model exhibited a significant separation of clusters among the three mouse groups in both cationic mode and anionic mode (Figures S7A and B). In the PLS‐DA model verification (the number of random replacement tests was 200 times), the replacement tests passed, and there was no overfitting phenomenon in the model (Figures S7C and D). A Venn diagram and volcano map showed that both CTX treatment and PPD‐M treatment resulted in dramatic alterations of metabolites, with a total of 101 metabolites (43 upregulated and 58 downregulated) in the CTX versus. CTR group and 117 metabolites (78 upregulated and 39 downregulated) in the PPD‐M versus CTX group (Figures S7E–G). These results suggest that gut microbiota regulated by PPD alters the composition of fecal metabolites.

**FIGURE 5:**
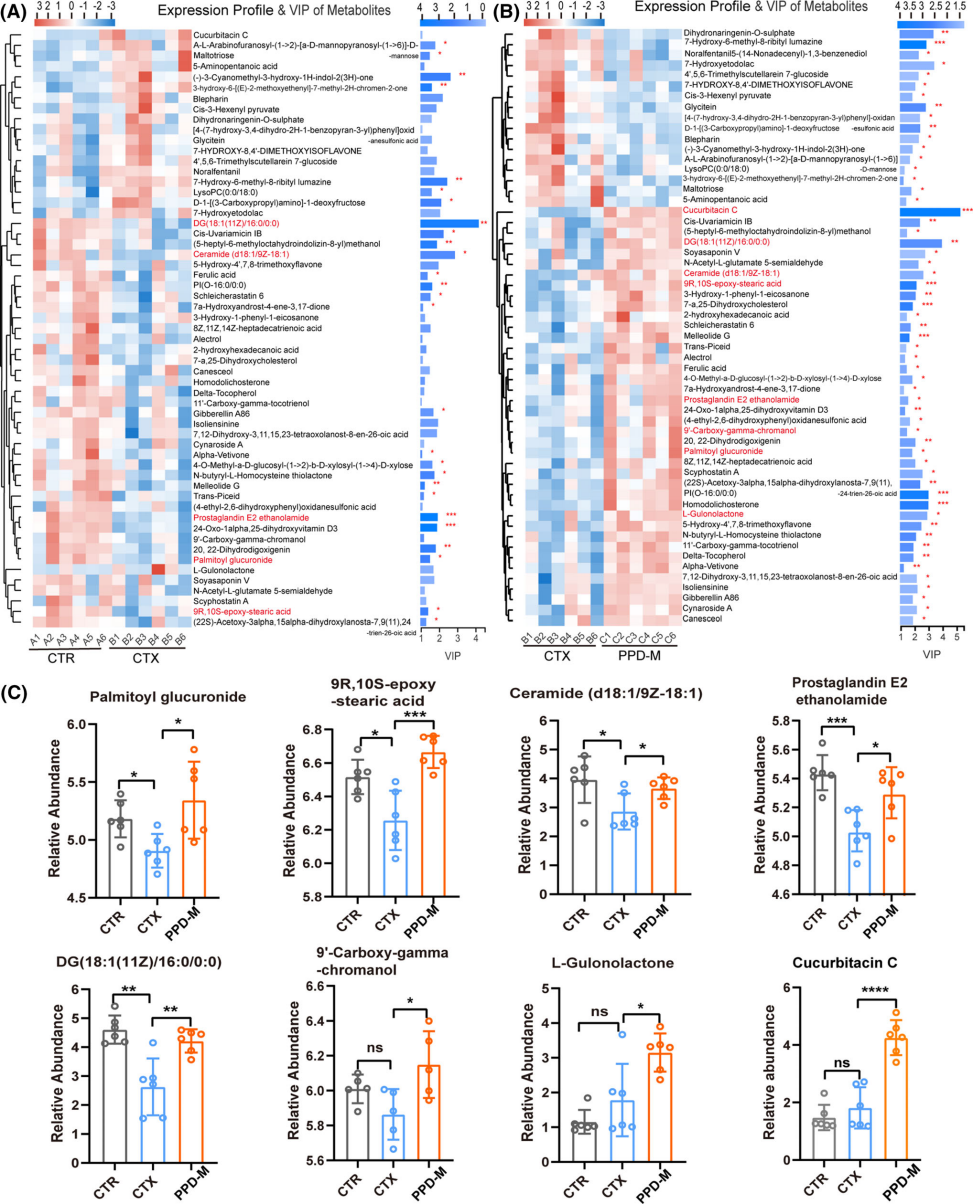
PPD induces changes in fecal metabolites. (A) and (B) Heat map of significantly altered metabolites in each sample. Metabolites with a variable importance in the projection (VIP) value of OPLS‐DA model > 1 and *p* values of Student's *t*‐test < 0.05 were considered to be significantly different. (*n* = 6, *p < 0.05, **p < 0.01, ***p < 0.001). (C) Metabolites with significantly different changes in the PPD‐M group and the CTX group (data expressed as the mean ± SD. *n* = 6, *p < 0.05, **p < 0.01, ***p < 0.001, ****p < 0.0001, “ns” means no significant difference.)

A heatmap was used to visualize the importance and expression trend of the differential metabolites in the CTX versus CTR group and PPD‐M versus CTX group with variable importance in the projection (VIP) value of multivariate statistical analysis and P values of one‐dimensional statistics (Figures 5A and B). Of note, the metabolites of 9R,10S‐epoxy‐stearic acid (ESA), ceramide (d18:1/9Z‐18:1), DG (18:1(11Z)/16:0/0:0), palmitoyl glucuronide (PG), and prostaglandin E2 ethanolamide (PEE) were significantly decreased in CTX‐treated mice, while PPD treatment dramatically reversed the CTX‐induced decrease in these five metabolites (Figure 5C). In addition, PPD treatment also significantly enhanced the levels of Cucurbitacin C, l‐gulonolactone and 9′ ‐carboxy‐gamma‐chromanol (CGC) (Figure 5C).

**FIGURE 6:**
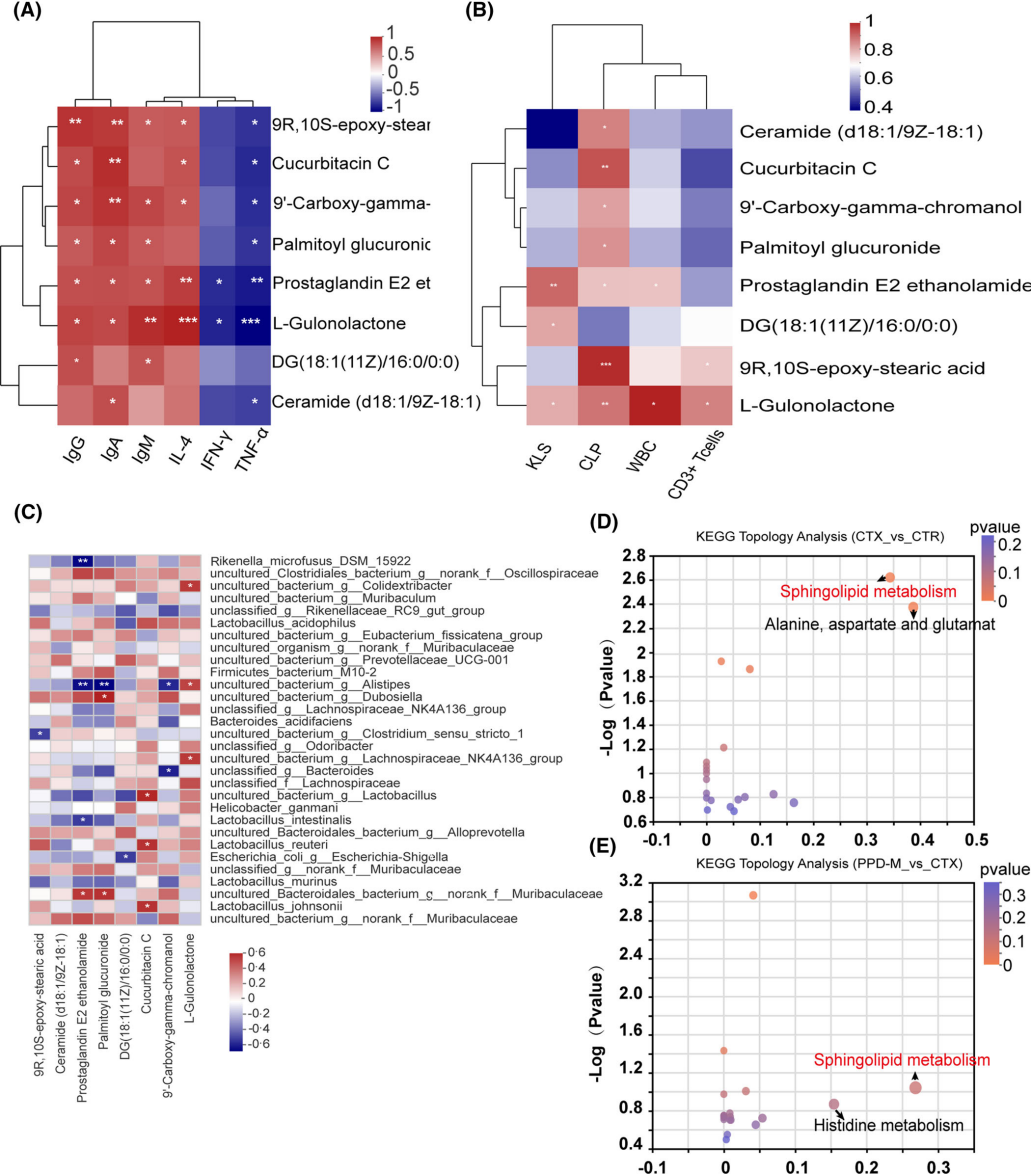
Functional analysis of differential metabolites. (A) Heat map of linear correlation analysis of differential metabolites with immunoglobulin and cytokines. (B) Heat map of linear correlation analysis of differential metabolites with immune indices (HSPCs, CLPs, WBCs, CD3^+^ T cells). (C) Heat map of linear correlation analysis between differential metabolites and gut microbiota. (D) KEGG Topology Analysis (CTX vs CTR); (E) KEGG Topology Analysis (PPD‐M vs. CTX). (Each bubble represents a KEGG pathway.)

Spearman correlation analysis showed that the abovementioned eight differential metabolites (ESA, ceramide, DG, PG, PEE, cucurbitacin C, l‐gulonolactone, and CGC) were all positively correlated with IgG, IgA, IgM, and IL‐4 but negatively correlated with IFN‐γ and TNF‐α (Figure 6A). Similarly, HSPCs, CLPs, WBCs, and CD3^+^ T cells were positively correlated with these 8 differential metabolites (Figure 6B). In addition, these metabolites were well correlated with many gut bacteria identified in the PPD‐M group (Figure 6C). Remarkably, cucurbitacin C was positively correlated with *uncultured_bacterium_g_Lactobacillus*, *Lactobacillus_reuteri*, and *Lactobacillus_johnsonii*, which was consistent with the fact that PPD treatment could significantly increase the abundance of *Lactobacillus* (Figure 3E), suggesting that cucurbitacin C may be a metabolite of *Lactobacillus*. Similarly, l‐gulonolactone was positively correlated with *uncultured_bacterium_g_Colidextribacter*, *uncultured_bacterium_g_Alistipes*, and *uncultured_bacterium_g_Lachnospiraceae_NK4A136_group* (Figure 6C). Figures 4G and H show that *Colidextribacter* was significantly positively correlated with IgG, IgM, IL‐4, WBCs and CD3^+^ T cells and negatively correlated with IFN‐γ and TNF‐α, and *g_Lachnospiraceae_NK4A136_group* was negatively correlated with IFN‐γ, suggesting that l‐gulonolactone may be a metabolite of *g_Colidextribacter* and *g_Lachnospiraceae_NK4A136_group*. The abovementioned results indicate that PPD promotes the production of immune‐related metabolites by remodeling the gut microbiota.

To further clarify the influence of PPD‐M on metabolic function in CTX‐induced immunosuppressed mice, the Kyoto Encyclopedia of Genes and Genomes (KEGG) pathway enrichment analysis was performed. The obtained results showed that two biological pathways (impact values>0.1, *p*<0.05) were significantly affected by CTX compared with the CTR group, and these changes were primarily linked to sphingolipid metabolism and alanine, aspartate and glutamate metabolism (Figure 6D). The comparison between the PPD‐M group and the CTX group showed that the sphingolipid metabolism pathway was also significantly affected by PPD‐M (impact values>0.1, *p*<0.05) (Figure 6E), indicating the importance of the sphingolipid metabolism pathway. According to the KEGG topology analysis, the main metabolite enriched in the sphingolipid metabolism pathway was ceramide (D18:1/9Z‐18:1). Figure 5 shows that CTX treatment significantly reduced the ceramide level, while PPD treatment perfectly restored the CTX‐induced ceramide reduction. These results illustrate that the sphingolipid metabolism pathway plays an important role in PPD‐M improvement of CTX‐induced immunosuppression.

## 3 DISCUSSION

The gut microbiota is strongly implicated in host physiology and pathophysiology.^26^ Gastrointestinal mucositis is one of the serious complications of chemotherapy and radiotherapy and affects approximately 50% of cancer patients.^27^ CTX has the side effect of damaging the intestinal mucosal barrier and causing dysregulation of gut microbiota.^14^ In this study, 16S rRNA gene sequencing was used to detect the gut microbiota of mice. LEfSe analysis showed that *g_Escherichia‐Shigella* was enriched in the CTX group, which has been identified as an opportunistic pathogen that can cause inflammation.^28^, ^29^ After PPD treatment, *Lactobacillus* was significantly enriched, and *o_Oscillospirales*, *g_Turicibacter*, and *g_Parabacteroides* were also abundant (Figure 3E). *Lactobacillus* is defined as a probiotic that can promote digestion and absorption, improve the function of the human gastrointestinal tract, increase beneficial intestinal bacteria, restore the balance of intestinal bacteria, and enhance human immunity and resistance.^30^ Butyrate is an important reference index for screening “the next generation of probiotics.” *Oscillospirales* can produce butyrate and other short‐chain fatty acids (SCFAs); thus, it is listed as a candidate for the next generation of probiotics.^31^ *Turicibacter* was positively correlated with enhanced immune functions,^32^ which was confirmed by the fact that the populations of *Turicibacter* in the gastrointestine were virtually nonexistent in both innate and adaptive immunodeficiency mice.^33^, ^34^ Therefore, in our study, CTX treatment led to the proliferation of pathogenic bacteria, while PPD‐M treatment increased the abundance of beneficial bacteria.

Broad‐spectrum ABX experiments showed that the gut microbiota plays an important role in PPD‐M enhancing immunity (Figures 4A–F). Spearman correlation analysis indicated that *Colidextribacter, f_Oscillospiraceae*, and *Oscillibacter* were significantly positively correlated with IgG, IgM and IL‐4 but negatively correlated with IFN‐γ and TNF‐α. Moreover, HSPC, WBC and CD3^+^ T cells were significantly positively correlated with *f_Lachnospiraceae, g_Colidextribacter, g_Dubosiella*, and *g_Alloprevotella* but negatively correlated with *g_Escherichia‐Shigella* (Figures 4G and H). Recent studies have reported that *Coldextribacter* metabolizes to produce inosine, which can enhance the efficacy of checkpoint blockade immunotherapy and has anti‐inflammatory effects.^35^, ^36^ *Lachnospiraceae* can protect mice against damage to the hematopoietic system and intestinal system caused by radiation and chemotherapy.^37^ *Dubosiella* also regulates metabolism in vivo, improving intestinal immunity and promoting the body's resistance to inflammatory diseases.^38^, ^39^ *Alloprevotella* has been shown to benefit the production of SCFAs.^40^

How does the gut microbiota influence the immunomodulatory function of PPD‐M? Untargeted metabolomic analysis of mouse fecal contents was used to explain this mechanism. CTX treatment decreased the levels of 9R,10S‐epoxy‐stearic acid, ceramide, DG, palmitoyl glucuronide, and prostaglandin E2 ethanolamide, while PPD‐M treatment dramatically reversed these decreases. In addition, PPD treatment also significantly enhanced the levels of cucurbitacin C, l‐gulonolactone and 9′ ‐carboxy‐gamma‐chromanol (Figure 5C). 9R, 10S‐epoxy‐stearic acid has anti‐inflammatory and preventive effects, as well as anticancer activity.^41^ Palmitoyl glucuronide is negatively correlated with ulcerative colitis.^42^ Prostaglandin E2 ethanolamide is a prostaglandin derivative that can reduce cytokine‐induced intestinal epithelial injury.^43^ l‐gulonolactone does not enhance immunity by itself, but it is an essential component of l‐gulono‐lactone oxidase (GLO), a critical enzyme in the vitamin C synthesis pathway, promoting the synthesis of vitamin C. Vitamin C has a variety of beneficial effects on the cellular functions of both the innate and adaptive immune systems.^44^ Cucurbitacin C itself has immune‐enhancing effects, and a recent study found that cucurbitacin C also has a variety of antitumor effects.^45^ In this study, Spearman correlation analysis showed that these 8 differential metabolites were positively correlated with IgG, IgA, IgM, IL‐4, HSPCs, CLPs, WBCs, and CD3^+^ T cells and negatively correlated with IFN‐γ and TNF‐α (Figures 6A and B), illustrating that these metabolites have immune‐enhancing and/or anti‐inflammatory effects. More importantly, these differential metabolites are closely related to the gut microbiota (Figure 6C). In particular, cucurbitacin C was positively correlated with *Lactobacillus_reuteri* and *Lactobacillus_johnsonii*, suggesting that cucurbitacin C may be a metabolite of *Lactobacillus*. l‐gulonolactone was positively correlated with *g_Colidextribacter*, *g_Alistipes*, and *g_Lachnospiraceae_NK4A136_group*, suggesting that l‐gulonolactone may be their metabolite. Therefore, we conclude that PPD‐M exerts its immunomodulatory function by promoting beneficial bacteria to produce immunity‐enhancing metabolites.

In addition, KEGG topology analysis showed that the sphingolipid metabolic pathway was significantly enriched in both the CTX versus CTR and PPD‐M versus CTX groups, and ceramide was the main metabolite of this pathway (Figures 6D and E). In CTX‐induced immunosuppressed mice, ceramide levels were significantly reduced, while PPD treatment perfectly restored this reduction (Figure 5). The sphingolipid metabolic pathway has become an important target for the treatment of many diseases. Currently, many bioactive sphingolipid analogs have been proven to be used for the treatment of immunity, metabolic disorders, and cancer, such as FTY720.^46^ Ceramide is a central hub of the sphingolipid metabolic pathway. Ceramide accumulation is considered to be a key step in mediating and regulating various immune cell functions such as regulation of the immune cell response to bacteria, viruses, and other foreign pathogens, production of cytokines, optimization of hematopoietic stem cell mobilization and homing, formation of NETs in neutrophils, and mediation of T cell responses during inflammation.^47^, ^48^ In this study, ceramide was positively correlated with CLP and IgA but negatively correlated with TNF‐α (Figures 6A and B), suggesting that ceramide was involved in the processes of promoting bone marrow hematopoiesis, enhancing immunity and inhibiting inflammation. In industry, microbial fermentation technology is a commonly used production method of ceramide,^49^ and a previous study has shown that gut‐derived sphingolipids produced by gut *Bacteroides* affect ceramide levels,^50^ suggesting that ceramide production is closely related to gut bacteria. In summary, these results demonstrate that the sphingolipid metabolic pathway may be a potential target of PPD for enhancing immunity in immunosuppressed mice.

**FIGURE 7:**
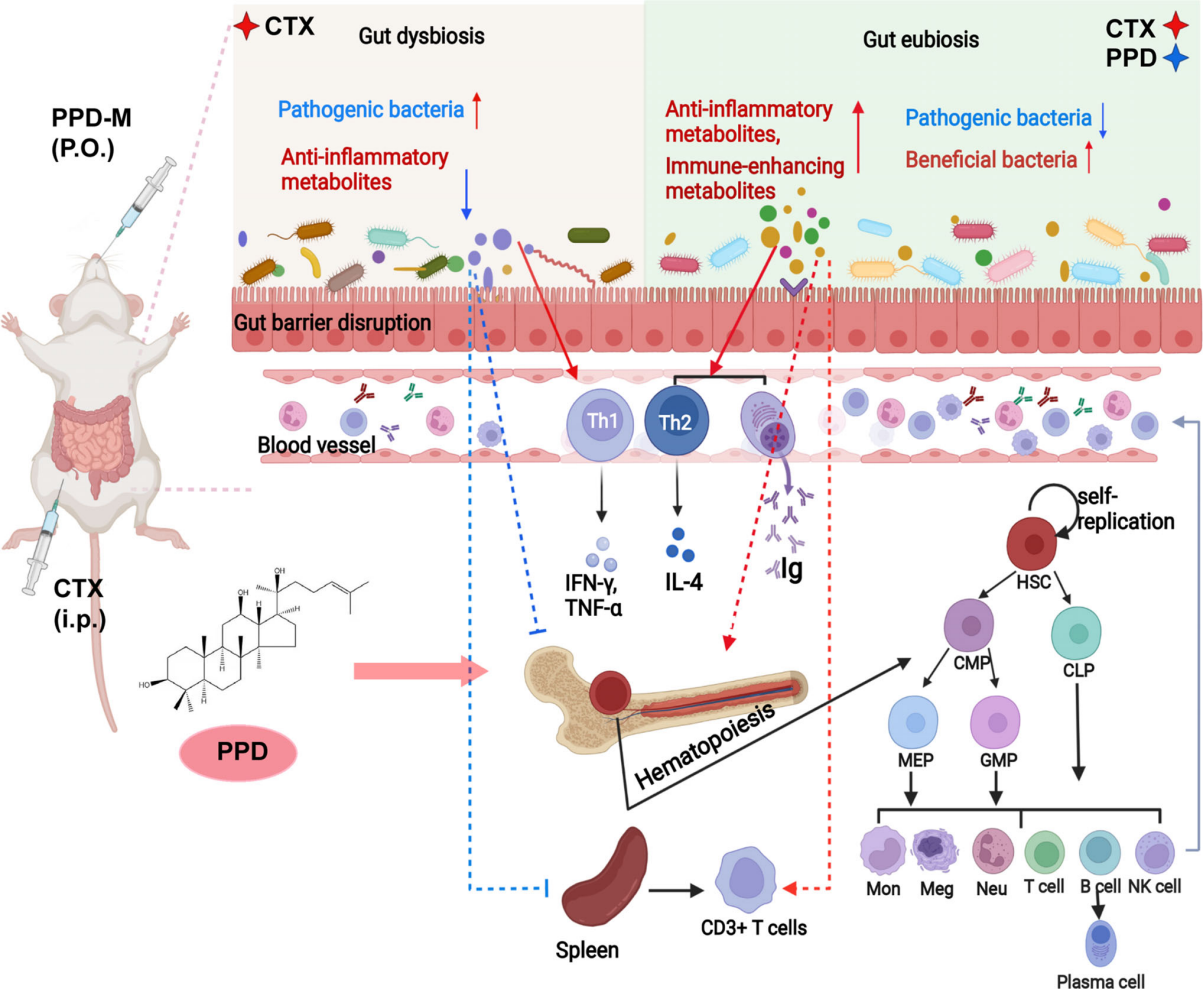
A schematic model showing the immunomodulatory mechanism of PPD in CTX‐induced immunosuppressed mice. CTX treatment leads to immunosuppression and gut microbiota dysbiosis in mice. PPD treatment can protect against CTX‐induced gut microbiota dysbiosis by increasing the abundance of beneficial bacteria and inhibiting the proliferation of pathogenic bacteria. The beneficial bacteria metabolize to produce immune‐enhancing metabolites and anti‐inflammatory metabolites, which contribute to the immune‐enhancing and anti‐inflammatory functions of PPD‐M in CTX‐induced immunosuppressed mice, including promoting bone marrow hematopoiesis to increase the number of peripheral blood white blood cells, proliferating splenic lymphocytes and eliminating inflammation. (The schematic was drawn with the help of Biorender.)

In conclusions, this work provides the first evidence that PPD promotes bone marrow hematopoiesis and improves immunity by regulating gut microbiota in an immunosuppressed mouse model. PPD‐M effectively ameliorated CTX‐induced immunosuppression in mice by promoting bone marrow hematopoiesis, increasing the number of peripheral blood WBCs, protecting peripheral lymphoid tissue and increasing the levels of immunoglobulin IgG and IgM in blood. More importantly, PPD‐M protected against CTX‐induced gut microbiota dysbiosis by increasing the abundance of beneficial bacteria and inhibiting the proliferation of pathogenic bacteria. When the gut microbiota was depleted, PPD‐M lost its ability to promote bone marrow hematopoiesis and enhance immunity. In addition, PPD‐M promoted the production of microbiota‐derived anti‐inflammatory and immune‐enhancing metabolites and the enrichment of the sphingolipid metabolic pathway, which contributed to the immune‐enhancing function of PPD‐M in immunosuppressed mice (Figure 7). Our study revealed a new mechanism by which PPD enhances immunity and provided a scientific basis for PPD as an immunomodulator. PPD has potential clinical applications in reducing the side effects of cancer chemotherapy.

## 4 MATERIALS AND METHODS

### 4.1 Animal and experimental design

ICR mice (5‐6 weeks old, 22 ± 2 g, male) used in this study were obtained from the Pengyue Experimental Animal Center (No. SCXK20190003, Jinan, China). For immunosuppressed mouse model preparation, 56 mice were randomly divided into two groups: the control (CTR) group and the CTX group. Mice in the CTX group were intraperitoneally injected with CTX (50 mg/kg) solution, and mice in the CTR group were intraperitoneally injected with the same volume of physiological saline. On the day before treatment with CTX and on the first, third, and sixth days after treatment with CTX, seven mice in each group were randomly removed for experimental testing at each time point.

To explore the immune improvement of PPD, 30 mice were randomly divided into five groups: the control group (CTR), CTX group, CTX+PPD low‐dose group (PPD‐L), CTX+PPD medium‐dose group (PPD‐M), and CTX+PPD high‐dose group (PPD‐H). PPD (HPLC ≥98%) was dissolved in vehicle containing 5% anhydrous ethanol, 0.5% Tween‐80 and 94.5% deionized water. The mice in the PPD groups were administered PPD by oral gavage at doses of 25 mg/kg/day (PPD‐L), 50 mg/kg/day (PPD‐M), and 100 mg/kg/day (PPD‐H) for 16 days. The mice in the CTR and CTX groups were given PPD vehicle for 16 days. On the 14th day, the mice in the CTX, PPD‐L, PPD‐M, and PPD‐H groups were given CTX (50 mg/kg) intraperitoneally, and the CTR group was administered an equivalent dose of saline. On the 17th day, the mice were sacrificed for fecal, blood, and organ samples for further testing.

### 4.2 Depletion of gut microbiota

Mice freely drank water with broad‐spectrum ABX, containing ampicillin (1 mg/ml), kanamycin (1 mg/ml), streptomycin (1 mg/ml), and vancomycin (0.5 mg/ml) (Meilunbio, China). The control group mice were also maintained with sterile water and sterile food during the experiment. Rectal feces were collected at 1 week after antibiotic administration and at the end of the experiment and then cultured in LB liquid medium or LB solid medium for 12 h. The absorbance and photographs were recorded.

### 4.3 Cytokine and immunoglobulin assays

Serum was collected from blood samples by centrifugation at 4000 rpm and 4°C for 15 min. The contents of TNF‐α, IFN‐γ, IL‐4, IgG, IgM, and IgA were measured by ELISA kits (Shanghai MLBIO Biotechnology, China).

### 4.4 Flow cytometry assay

The spleen was ground and filtered through a 70 μm nylon mesh to obtain a single‐cell suspension. Lymphocytes were isolated using a Mouse Spleen Lymphocyte Isolation Kit (Solarbio, China). The lymphocytes were washed twice with phosphate‐buffered saline (PBS) and resuspended. Then, antibodies against CD3‐PE, CD4‐APC, and CD8a‐FITC (BioLegend, USA) were added to the cell suspension in sequence. After gentle mixing, the mixture was incubated for 30 min in a dark room at 4°C. The percentages of CD3^+^ T cells, CD4^+^ T cells and CD8a^+^ T cell subsets were analyzed by flow cytometry (BD Canto II, USA).

Bone marrow was collected by flushing femurs with RPMI 1640 medium. Red blood cells were removed from single‐cell suspensions with RBC lysis buffer (Solarbio, China). One milliliter of cell suspension was added to Fixable Dye eFluor 780 (Thermo Fisher Scientific, USA) to isolate dead cells. After gentle swirling, the cells were incubated for 30 min in the dark at 4°C. After washing with PBS, the cells were resuspended. The treated cells were analyzed for hematopoietic stem/progenitor cells (HSPCs). In short, lineage marker‐negative cells (Lin^−^) were first separated using Hematopoietic Lineage Antibody Cocktail FITC (Thermo Fisher Scientific, USA) (containing antibodies against CD3, CD45R, CD11b, Gr‐1, and Ter‐119). Lin^−^ cells were then further stained with Sca‐1‐PerCP/Cyanine5.5, c‐Kit‐APC, IL‐7Rα‐PE/Cyanine7, CD34‐Brilliant Violet 421™, and CD16/32‐PE (BioLegend, USA). Populations of KLS^+^ cells (Lin^−^ c‐Kit^+^ Sca‐1^+^, HSPCs), KLS^−^ cells (Lin^−^ c‐Kit^+^ Sca‐1^−^), CLPs (KLS^+^ IL‐7Rα^+^), CMPs (KLS^−^ CD34^+^ IL‐7Rα^−^ CD16/32^−^), GMPs (KLS^−^ CD34^+^ IL‐7Rα^−^ CD16/32^+^), and MEPs (KLS^−^ IL‐7Rα^−^ CD34^−^ CD16/32^−^) were analyzed by flow cytometry. Data were analyzed with FlowJo software (Tree Star, USA), and the loop‐gate strategy is shown in Supplementary information Figure S2.

### 4.5 Histological analysis

The thymus, spleen, and thighbones were prepared for histological analysis according to previous methods.^12^ Briefly, the spleens and thymus were fixed in 4% paraformaldehyde, embedded in paraffin, and sliced into 5‐μm‐thick sections. Thighbones also need to be decalcified after fixation with 4% paraformaldehyde. To observe the changes within the organization, the tissues were stained with hematoxylin and eosin (HE) after deparaffinization. Images were obtained with a light microscope (OLYMPUS BX53, Japan).

### 4.6 Gut microbiota analysis

Fecal samples from mice in the CTR group, CTX group and PPD‐M group were collected aseptically, quickly frozen with liquid nitrogen, and then stored in the refrigerator at −80°C. DNA was extracted for gut microbiota diversity analysis. Fecal sample DNA extraction, PCR amplification and sequencing, and processing of sequencing data were performed at Majorbio Biotech (Shanghai, China). The data were analyzed using the Majorbio Cloud Platform (www.majorbio.com).

### 4.7 Fecal metabolite assessment

Fecal samples of mice in the CTR group, CTX group, and PPD‐M group were frozen at −80°C prior to LC‒MS analysis. Metabolite sample preparation and LC‒MS analysis parameters were conducted by Majorbio Biotech (Shanghai, China). Detailed steps are available in the Supplementary information. The data were analyzed using the Majorbio Cloud Platform (www.majorbio.com).

### 4.8 Statistical analysis

For two‐group comparisons, an unpaired two‐tailed Student's *t*‐test was applied. For significantly changing bacterial lists, we used the online Majorbio Cloud Platform to perform the Wilcoxon rank‐sum test, and the *p* value was based on a two‐tailed FDR‐corrected test. The significance level was set to 0.05, and the 0.95 confidence intervals were calculated through the bootstrap algorithm. For comparisons of more than two groups, one‐way ANOVA or two‐way ANOVA followed by Dunnett's test was performed. All data with error bars are represented as the mean ± SD, and *p* < 0.05 was considered statistically significant. Most of the data were analyzed in GraphPad Prism (Graph Pad Software Inc., USA).

## AUTHOR CONTRIBUTION

Y. C. and X. P. designed the study and wrote the article. Y. C., B. L., and W. L. performed the experiments and analyzed the data. L. Y., H. L., and C. L. assisted with the animal work. F. G. and X. G. provided useful suggestions. Z. S. helped with the data analysis. J. L. interpreted the results and revised the manuscript. All authors have read and approved the final manuscript.

## CONFLICT OF INTEREST STATEMENT

The authors declare no conflicts of interest.

## ETHICS STATEMENT

All animal experiments were approved by the Laboratory Animal Ethics Committee of Binzhou Medical University and complied with the national and international guidelines for the Care and Use of Laboratory Animals.

## Supporting information

Supporting Information

## Data Availability

The data supported the results in this study are available from the corresponding author upon reasonable request.
