# Supplementary material for: Protopanaxadiol manipulates gut microbiota to promote bone marrow hematopoiesis and enhance immunity in cyclophosphamide‐induced immunosuppression mice
Source: MedComm (2020). 2023 Feb 23;4(2):e222. doi: 10.1002/mco2.222 (PMC9950037; doi:10.1002/mco2.222)
Supplement: Supplementary file 1 — Supporting Information [file MCO2-4-e222-s001.pdf]

Supplementary Information

Protopanaxadiol promotes bone marrow hematopoiesis and enhances immunity by regulating gut microbiota in cyclophosphamide-induced immunosuppression mice

Yuru Cao[1,2,#], Ben Liu[2,#], Wenzhen Li[1], Feng Geng[1], Xue Gao[1], Lijun Yue[1], Huiping Liu[1], Zhenguo Su[2, *], Junhong Lü [1,3,4, *], and Xiaohong Pan[1, *]

[1] *School of Pharmacy, Binzhou Medical University, Yantai264003, China;*

[2] *Yantai Affiliated Hospital of Binzhou Medical University, Yantai264003, China;*

[3] *Shanghai Advanced Research Institute, Chinese Academy of Sciences, Shanghai 201203, China;*

[4] *Jinan Microecological Biomedicine Shandong Laboratory, Jinan 250000, China.*

[*]Corresponding authors (Xiaohong Pan, email: panxiaohong@bzmc.edu.cn; Junhong Lü, email: lvjh@sari.ac.cn; Zhenguo Su, email: szg68@126.com)

#Yuru Cao and Ben Liu contributed equally to this work.

Construction and evaluation of the immunosuppressed mouse model

According to previous reports [1, 2], 50 mg/kg of CTX was selected in this study. Figure S1a shows the protocol for constructing the mouse immunosuppression model. The weight of the mice decreased significantly within 1-6 days after CTX injection (Fig. S1b). The spleen index and thymus index decreased significantly within 1-3 days after CTX injection, with the most obvious decrease on the 3rd day and almost recovered on the 6th day (Fig. S1c and d). The number of peripheral blood white blood cells (WBCs) decreased significantly within 1-6 days after CTX injection, and the decrease was most obvious on the 3rd day (Fig. S1e). The results of WBC classification and counting showed that Lymphocyte (Lym) had the largest proportion of changes in peripheral blood WBC before and after CTX injection (Fig. S1f), which also confirmed the previous report that Lymphocyte line was one of the most sensitive cell lines to CTX [3]. These results proved that the immunosuppressed mouse model was successfully constructed. Since the inhibitory effect of CTX on various immune indexes was the best on the 3rd day after CTX injection, the follow-up experiments in this study selected the 3rd day of CTX injection (Day 3) as the time point to detect various indexes of mice.

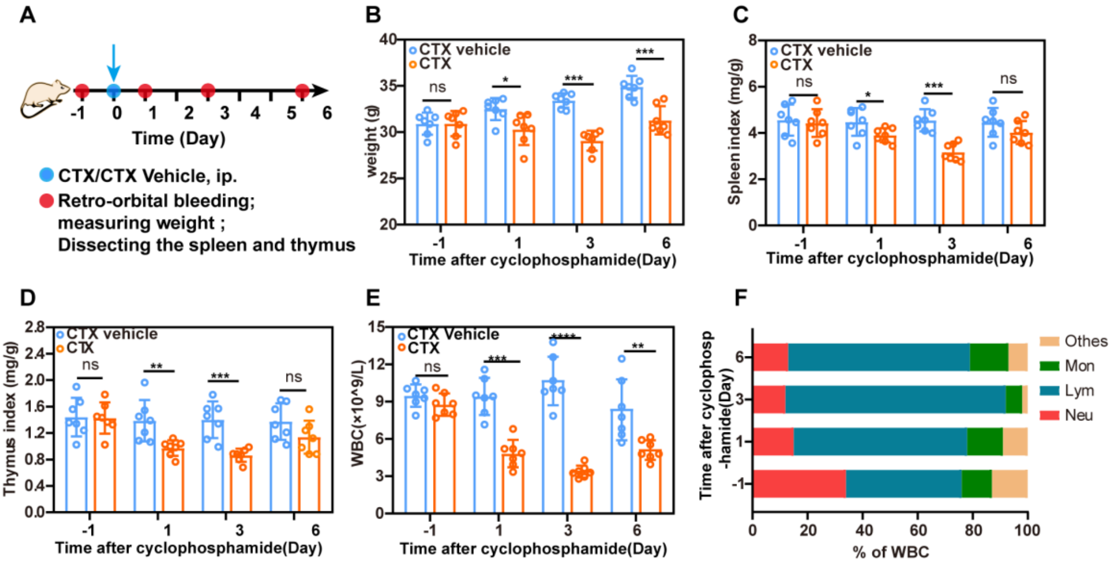

Fig. S1 Construction and evaluation of the immunosuppressed mouse model. (A) On day 0, mice

were intraperitoneally injected with CTX (50 mg/kg) or CTX vehicle, and various immune indexes of mice were detected on day -1 (before the injection of CTX), 1, 3, and 6. (B) The weight of mice was recorded in the control group and CTX group. (C) The spleen index was recorded in the control group and CTX group. (D) The thymus index was recorded in the control group and CTX group. (E) Changes of WBC count in the peripheral blood of mice in the control group and CTX group. (F) The ratio of lymphocytes (Lym), monocytes (Mon) and neutrophils (Neu) to white blood cells in the peripheral blood of mice before and after CTX injection (Othes refers to the unclassified cells in white blood cells). (n =7, *P < 0.05, **P < 0.01, ***P < 0.001, **** P < 0.0001).

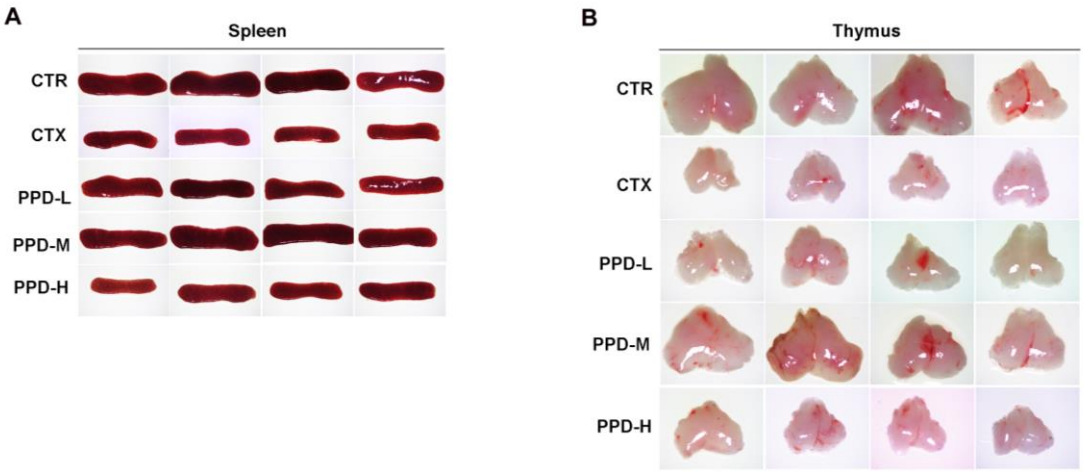

Fig. S2 Morphological observation of spleen (A) and thymus (B) of mice in each group under stereoscope

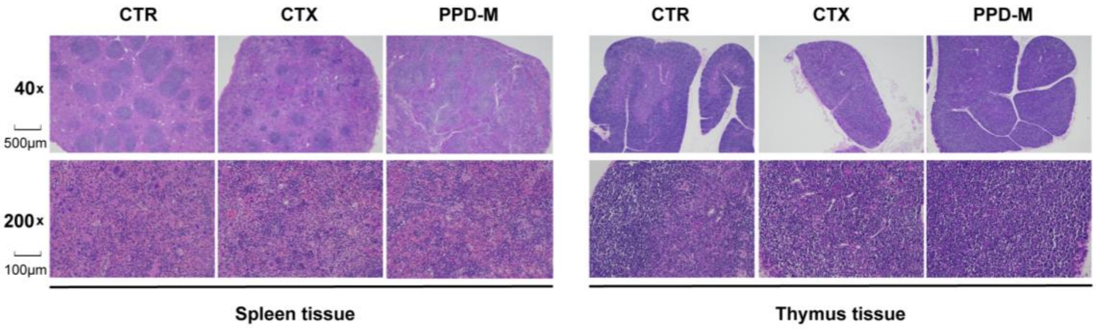

Fig. S3 HE staining of spleen tissue and thymus tissue.

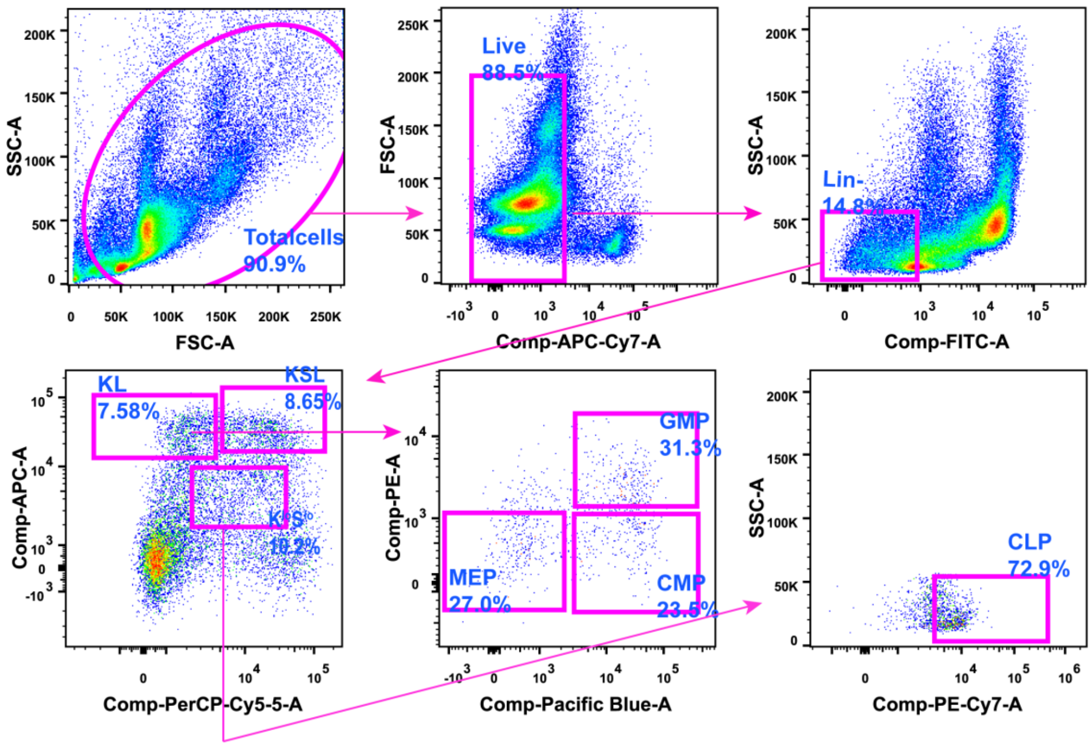

Fig. S4 Illustration of the flow cytometry gating strategy for identifying KSL, GMP, CMP, MEP and CLP.

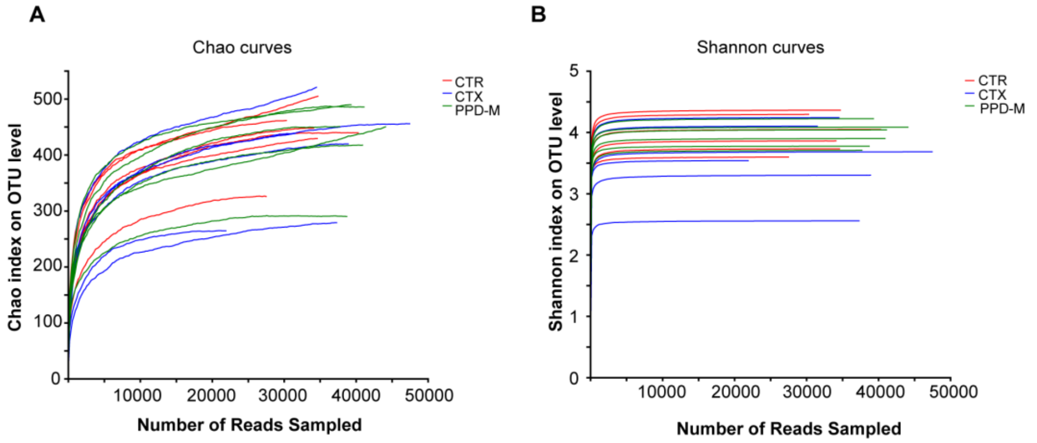

Fig. S5 The dilution curves of Chao diversity index (A) and Shannon diversity index (B).

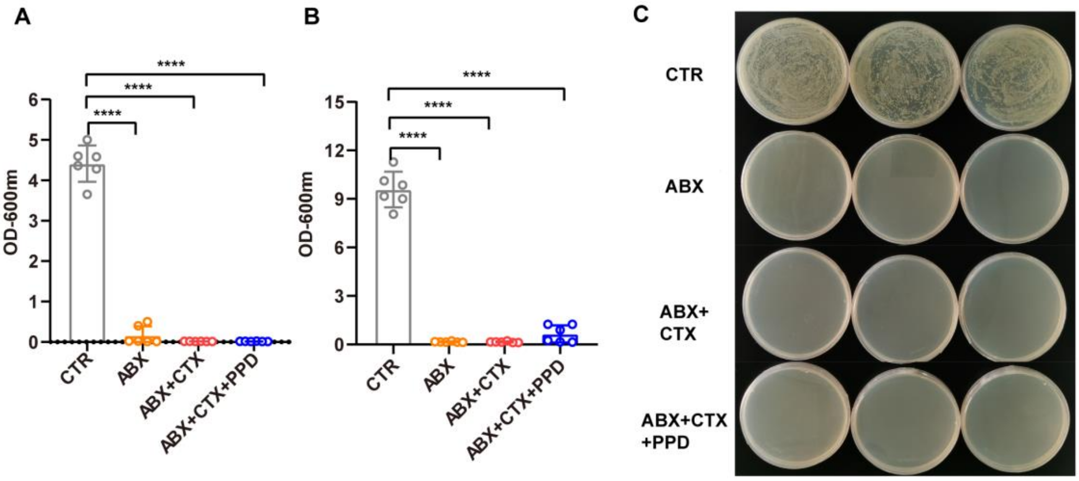

Fig. S6 Detection of intestinal bacteria in broad-spectrum antibiotics (ABX)-treated mice. At the day 0 (7 days after ABX treatment or at the beginning of treatment with PPD) (A) and the day 16 (at the end of the experiment) (B), mice feces were collected in a sterile environment, and then dissolved in normal saline at a concentration of 0.05 g/mL. Then, 20 μL feces solution was cultured in LB liquid medium for 12 h, and absorbance was measured. (C) 80 μL feces solution was cultured in LB solid medium for 12 h, and photos were harvested. (Data expressed as the Mean ± SD, n=6, *****P*<0.001)

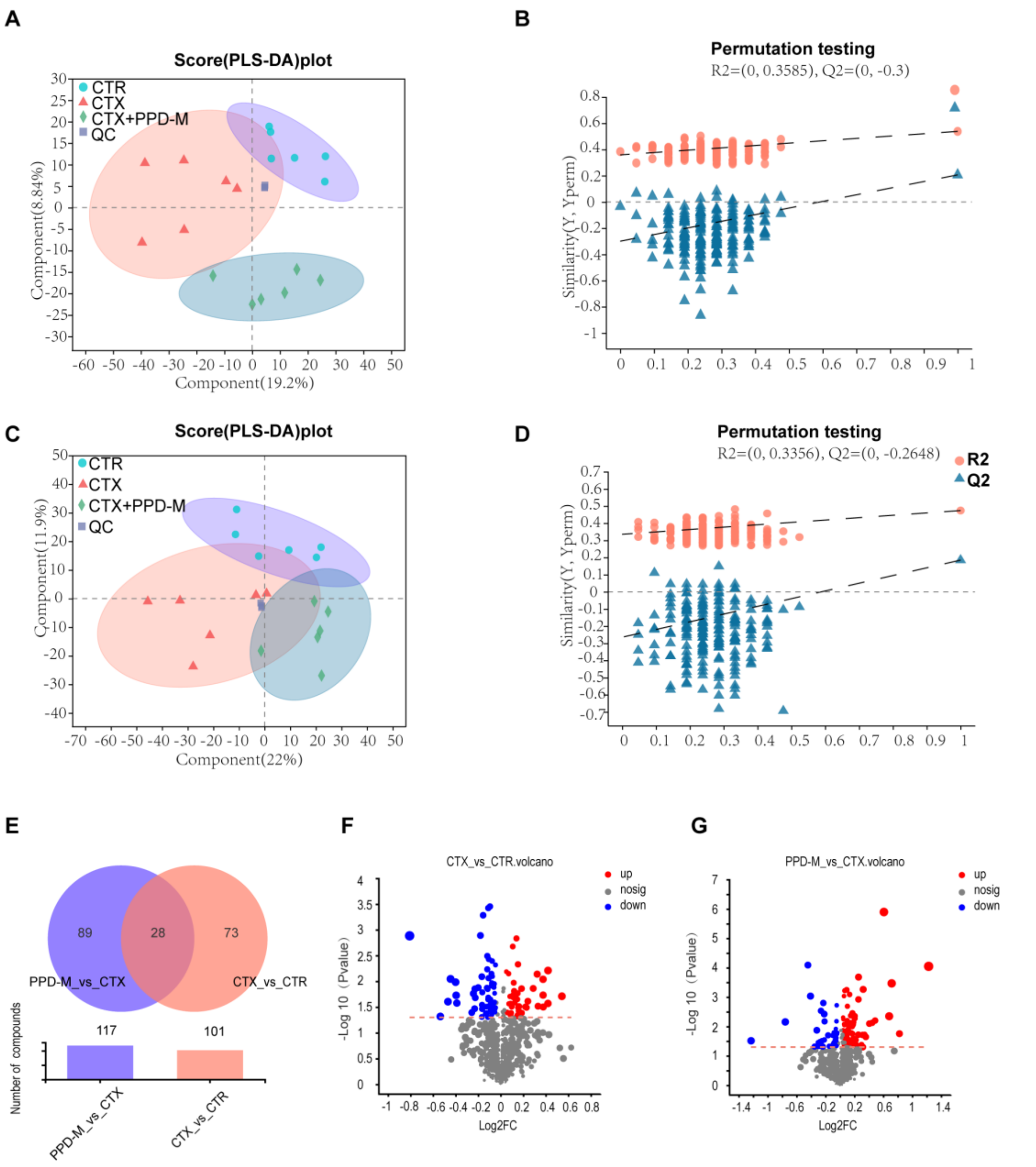

Fig. S7 Sample comparative analysis. PLS-DA analysis in cationic mode (A) and anionic mode (B). (C) PLS-DA model validation (cationic model). (D) PLS-DA model validation (anionic mode). (E) Venn diagram. (F) Volcanic map of differential metabolites in CTX *vs* CTR group. (G) Volcanic map of differential metabolites in PPD-M *vs* CTX group.

References

1. Huang J, Huang J, Li Y, et al. Sodium Alginate Modulates Immunity, Intestinal Mucosal Barrier Function, and Gut Microbiota in Cyclophosphamide-Induced Immunosuppressed BALB/c Mice. *J Agric Food Chem*. 2021;69(25):7064-7073.

2. Bai RB, Zhang YJ, Fan JM, et al. Immune-enhancement effects of oligosaccharides from Codonopsis pilosula on cyclophosphamide induced immunosuppression in mice. *Food Funct*. 2020;11(4):3306-3315.

3. Dale DC, Fauci AS, Wolff SM. The effect of cyclophosphamide on leukocyte kinetics and susceptibility to infection in patients with Wegener's granulomatosis. *Arthritis Rheum*. 1973; 16 (5): 657-664.
